# Occlusion preconditioned mice are resilient to hypobaric hypoxia-induced myocarditis and arrhythmias due to enhanced immunomodulation, metabolic homeostasis, and antioxidants defense

**DOI:** 10.3389/fimmu.2023.1124649

**Published:** 2023-02-15

**Authors:** Gabriel Komla Adzika, Richard Mprah, Ruqayya Rizvi, Adebayo Oluwafemi Adekunle, Marie Louise Ndzie Noah, Prosperl Ivette Wowui, Seyram Yao Adzraku, Joseph Adu-Amankwaah, Fengli Wang, Yuwen Lin, Lu Fu, Xiaomei Liu, Jie Xiang, Hong Sun

**Affiliations:** ^1^ Department of Physiology, Xuzhou Medical University, Xuzhou, Jiangsu, China; ^2^ Department of Clinical Medicine, Xuzhou Medical University, Xuzhou, Jiangsu, China; ^3^ Department of Hematology, Key Laboratory of Bone Marrow Stem Cell, The Affiliated Hospital of Xuzhou Medical University, Xuzhou, China; ^4^ Department of Rehabilitation Medicine, The Affiliated Xuzhou Rehabilitation Hospital of Xuzhou Medical University, Xuzhou, Jiangsu, China; ^5^ Jiangsu Key Laboratory of New Drug Research and Clinical Pharmacy, Xuzhou Medical University, Xuzhou, Jiangsu, China; ^6^ Jiangsu Key Laboratory of Immunity and Metabolism, Department of Pathogen Biology and Immunology and Laboratory of Infection and Immunity, Xuzhou Medical University, Xuzhou, Jiangsu, China

**Keywords:** hypobaric hypoxia, myocarditis, myocardial remodeling, arrhythmias, remote ischemic preconditioning, immunomodulation, metabolic homeostasis, antioxidant responses

## Abstract

**Background:**

Sea-level residents experience altitude sickness when they hike or visit altitudes above ~2,500 m due to the hypobaric hypoxia (HH) conditions at such places. HH has been shown to drive cardiac inflammation in both ventricles by inducing maladaptive metabolic reprogramming of macrophages, which evokes aggravated proinflammatory responses, promoting myocarditis, fibrotic remodeling, arrhythmias, heart failure, and sudden deaths. The use of salidroside or altitude preconditioning (AP) before visiting high altitudes has been extensively shown to exert cardioprotective effects. Even so, both therapeutic interventions have geographical limitations and/or are inaccessible/unavailable to the majority of the population as drawbacks. Meanwhile, occlusion preconditioning (OP) has been extensively demonstrated to prevent hypoxia-induced cardiomyocyte damage by triggering endogenous cardioprotective cascades to mitigate myocardial damage. Herein, with the notion that OP can be conveniently applied anywhere, we sought to explore it as an alternative therapeutic intervention for preventing HH-induced myocarditis, remodeling, and arrhythmias.

**Methods:**

OP intervention (6 cycles of 5 min occlusion with 200 mmHg for 5 min and 5 min reperfusion at 0 mmHg – applying to alternate hindlimb daily for 7 consecutive days) was performed, and its impact on cardiac electric activity, immunoregulation, myocardial remodeling, metabolic homeostasis, oxidative stress responses, and behavioral outcomes were assessed before and after exposure to HH in mice. In humans, before and after the application of OP intervention (6 cycles of 5 min occlusion with 130% of systolic pressure and 5 min reperfusion at 0 mmHg – applying to alternate upper limb daily for 6 consecutive days), all subjects were assessed by cardiopulmonary exercise testing (CPET).

**Results:**

Comparing the outcomes of OP to AP intervention, we observed that similar to the latter, OP preserved cardiac electric activity, mitigated maladaptive myocardial remodeling, induced adaptive immunomodulation and metabolic homeostasis in the heart, enhanced antioxidant defenses, and conferred resistance against HH-induce anxiety-related behavior. Additionally, OP enhanced respiratory and oxygen-carrying capacity, metabolic homeostasis, and endurance in humans.

**Conclusions:**

Overall, these findings demonstrate that OP is a potent alternative therapeutic intervention for preventing hypoxia-induced myocarditis, cardiac remodeling, arrhythmias, and cardiometabolic disorders and could potentially ameliorate the progression of other inflammatory, metabolic, and oxidative stress-related diseases.

## Introduction

Sea-level residents suffer from altitude sickness when they hike or visit altitudes above ~2,500 m due to the hypobaric hypoxia (HH) conditions at such places. Altitude sickness typically presents clinical manifestations such as shortness of breath, headache, dizziness, tiredness, mental confusion, and loss of appetite (1). Meanwhile, recent studies have shown that besides the aforementioned symptoms, individuals experiencing altitude sickness have underlying myocarditis and arrhythmias that were either induced or aggravated by HH (2, 3). Evidently, HH has been shown to drive cardiac inflammation in both ventricles by inducing maladaptive metabolic reprogramming of macrophages which evokes hypersecretion of the proinflammatory mediator – inducible nitric oxide synthase (iNOS) and cytokines (C-Reactive Proteins, Interleukin (IL)-1β and IL-18). HH-induced hyperactive proinflammatory responses expedite adverse cardiac remodeling by activating and sustaining fibrosis cascades, ultimately resulting in heart failure and sudden cardiac death (4, 5).

Therapeutic approaches developed against altitude sickness over the years have mainly been preventive interventions targeted at circumventing or mitigating the adverse outcomes of HH exposure. Notably, the use of salidroside (a phenylethanoid glycoside found in *Rhodiola* genus plants) and altitude preconditioning (AP) (as known as intermittent HH preconditioning) prior to visiting high altitudes have been extensively shown to exert cardioprotective effects (6, 7). The efficacies of salidroside and AP interventions have been attributed to their abilities to decrease reactive oxygen species (ROS), induce adaptive regulation of antioxidants and anti-inflammatory-related pathways as well as enhance tissue oxygenation to prevent necrosis and apoptosis of cardiomyocytes (6–9). However, the availability of *Rhodiola* plants or salidroside is geographically limited to Europe, North America, and low-Arctic to high-temperature regions of Asia (10). Similarly, hypoxia chambers for AP are inaccessible/unavailable to the majority of the population, and the intervention cannot be applied at one’s convenience before hiking or visiting high altitudes.

Meanwhile, remote ischemic preconditioning [hereafter referred to as occlusion preconditioning (OP)] has been extensively demonstrated to prevent hypoxia-induced cardiomyocyte damage by triggering endogenous cardioprotective cascade (11–13). These generally positive outcomes of OP have encouraged its application in clinical trials and settings to reduce the severity of ischemic injuries and myocardial damage, even though the underlying mechanisms of the intervention are still being elucidated.

Here, with the notion that OP can be conveniently applied anywhere, we sought to explore it as an alternative therapeutic intervention for preventing HH-induced myocarditis and cardiac arrhythmia. Herein, we demonstrate the cardioprotective potentials of OP in HH by comparing its impact on cardiac electric activity, hypertrophy and injury, immunoregulation, oxidative stress responses, and behavioral outcomes, with AP’s in HH. Also, we showed that OP enhances respiratory and oxygen-carrying capacity in humans. In addition, numerous studies have shown that the β_2_-adrenergic receptor (β_2_AR) confers cardioprotection in stressful conditions, including hypoxia (14, 15). Hence, we utilized β_2_AR-knockout (β_2_AR-KO) mice and uncovered that β_2_AR is involved in mediating OP-induced cardioprotection in HH. These findings illustrate OP as a potent alternative therapeutic intervention for preventing hypoxia-induced myocarditis and as well suggest its potential to ameliorate other oxidative stress-related diseases.

## Methods

### Experimental animal model protocols

Eight- to twelve-week-old wild-type (Adrb2^+/+^) and β_2_AR-knockout (Adrb2^-/-^) FVB male mice were used in this study. The mice were kept and fed in a hypoxia chamber (Guizhou Fenglei Aviation Machinery Co., Ltd., Guizhou, China: FLYDWC50-IIA), and hypobaric hypoxia (HH) was induced by increasing altitude to 3000 m for 10 min, then to 4500 m for 10 min, followed by 5500 m for 20 min before finally increasing to 6000 m altitude for 7 days. Mice in the control group were kept and fed in a normobaric normoxia (NN) environment at sea level (with ambient oxygen percentage) for 7 days.

To explore the therapeutic potentials of limb occlusion ischemic preconditioning, hair was removed from mice’s hindlimbs. Limb occlusion preconditioning (OP) was performed by applying a 200 mmHg pressure tourniquet for 5 min and allowing 5 min reperfusion at 0 mmHg. Six cycles of OP were performed daily on alternate hindlimbs for 7 days. Next, the mice were randomized into two groups; the first group, (OP) mice, were sacrificed, and the second group was exposed to HH stepwise as previously described for 7 days. The latter group was designated as OP prior to HH exposure (OPHH) (**Supplementary Figure 1A**). Additionally, we sought to compare the experimental outcomes from OP and OPHH with altitude preconditioning (AP) prior to HH exposure (APHH) models; hence, AP was done by exposing wild-type FVB to HH at 3500 altitudes for 30 min daily, for 7 days. Afterward, the mice were randomized into two groups; the first group (AP) mice were sacrificed, and the second group (APHH) mice were exposed to HH in a stepwise manner as previously described for 7 days (**Supplementary Figure 1B).**

At the end of all experimental models, electrocardiography (EKG) data acquisitions were performed with PowerLab (ADInstruments, North America), and the mice were euthanatized by cervical dislocation. Hearts were excised, quickly wet-weighed for morphometric analysis, and processed for further investigations. The performed experiments were approved by the Experimental Animal Centre of Xuzhou Medical University and the Animal Ethics Committee of the Medical University (permit number: xz11- 12541) and conform to the Guide for the Care and Use of Laboratory Animals published by the US National Institutes of Health (NIH Publication, 8th Edition, 2011).

### Electrocardiography

Electrocardiography (EKG) data acquisitions were performed with the 3-lead monopolar needle electrode from PowerLab systems (ADInstruments, North America), as previously described (16).

### Assays

#### Enzyme-linked immunosorbent assay (ELISA)

Myocardia lysates were used to examine the concentration of proinflammatory (iNOS, IL-1β, and IL-18) and anti-inflammatory biomarkers (Arg-1, IL-10, and TGF-β) and cardiac hypertrophy/injury markers (ANP and BNP). Sera were used to assess cardiac troponin I (cTnI) and C-reactive protein (CRP) concentrations. IL-1β (JL18442; Jianglai Bio. Tech), IL-18 (JL20253; Jianglai Bio. Tech.), iNOS (JL20675; Jianglai Bio. Tech.), IL-10 (JL20242; Jianglai Bio. Tech.), TGF-β (JL13959; Jianglai Bio. Tech.), Arg-1 (JL13668; Jianglai Bio. Tech.), ANP (JL20612; Jianglai Bio. Tech.), BNP (JL12884; Jianglai Bio. Tech.), CRP (JL13196; Jianglai Bio. Tech.) and cTnI (JL31923; Jianglai Bio. Tech.) ELISAs were done in triplicates and as per the manufacturer’s instructions.

#### NAD/NADH content assay

Using equal weights (0.1 g) of myocardia, the coenzyme I NAD/NADH contents were assessed using assay kits (BC0310; Solarbio) and following the manufacturer’s instructions.

#### Total antioxidant capacity assay

Equal weights (0.1 g) of myocardia were used to evaluate the total antioxidant capacity (T-AOC). Assay kits (BC1310; Solarbio) were used according to the manufacturer’s instructions.

### Behavioral assessments

#### Open field test (OFT)

Locomotor activity and exploratory and anxiety-related behavior of the mice were examined before and after preconditioning or exposure to HH or NN by using the OFT apparatus. Briefly, the apparatus consists of a squared box (50cm x 50 cm) with its base divided into 9 squares; 1 central (zone C), 4 corners (zone B), and 4 peripheries (zone A). Before the initial and subsequent tests, fecal pellets or urine were cleaned, and the chamber was wiped-dry with 95% ethanol to remove any clues and scent left by the last tested mouse. The mice were individually placed in zone C and left undisturbed to explore for 5 min while their locomotion activities were tracked with video tracking software (ANY-maze version 7.00).

#### Elevated plus maze (EPM)

Utilizing the EPM apparatus, anxiety-related behaviors were examined before and after preconditioning or exposure to HH or NN. In brief, the EPM consists of a plus (+) shaped apparatus with a central point, two opposite arms enclosed, and the other opposite arms opened. Before the initial and subsequent tests, fecal pellets or urine were cleaned, and the arms were wiped-dry with 95% ethanol to remove any clues and scent left by the last tested mouse. During testing, the plus maze was elevated ~ 1 m from the floor, and each mouse was placed at the central point of the open and closed arms, with their head facing the open arm. The mice were allowed to explore the maze for 5 min while their locomotion activities and entries into either arm were tracked and recorded by video tracking software (ANY-maze version 7.00).

### Myocardial macrophage isolations

Mice were euthanized by cervical dislocation; hearts were exposed and perfused-blanch with iced-cold PBS through the right and left ventricle by using a 5 mL syringe and 25 G needle. Hearts were then transferred in 12-well plates containing 1 mg/mL collagenase IV (Gibco™: 17104019) in 3 mL Hanks’ Balanced Salt Solution (HBSS) kept on ice and minced with sterile scissors. Minced myocardia were digested for 45 min at 37°C on a shaker (50 rpm). Next, the plates were vortexed, kept on ice, and new Pasteur pipettes were used to dissociate cells mechanically. Obtained suspensions were filtered through 35 μm strainers into 15 ml tubes containing 10 ml cold HBSS, centrifuged at 1500 rpm for 5 min, and the supernatants were discarded. Red blood cells in the pellet were hemolyzed with ACK buffer (Gibco™: A1049201) and washed twice with PBS. Myocardial macrophage phenotypes were identified and sorted with FACS (BD FACSAria™ III) after resuspension and incubation with Fc Blocker (Invitrogen; 14-9161-73; 1:100), PE-Cy5 anti-CD45 (BD Pharmingen™; 553082; 1:100), APC anti-F4/80 (BioLegend; 123116; 1:100), FITC anti-CD11b (BioLegend; 101206; 1:100), PerCP anti-CD86 (BioLegend; 105028; 1:100) and PE anti-CD206 (BioLegend; 141706; 1:100).

### Histology, immunohistochemistry, and biochemical staining

#### Wheat germ agglutinin (WGA) staining

Cryopreserved heart sections were fixed with 4% formaldehyde for 30 min at room temperature (RT), washed thrice with PBS, and primed with HBSS for 15 min. Next, without permeabilization, the myocardial sections were incubated with WGA staining (Thermo Fisher Scientific; W11261) in the dark for 10 min at RT – followed by three times wash with PBS and DAPI counterstaining. Imaging was done at X60 magnification, and ImageJ (1.52a version; National Institute of Health USA) was used to assess cardiomyocyte surface area.

#### Masson’s trichrome staining

Myocardial sections were trichrome stained according to the manufacturer’s (Solarbio; G1340) instructions. Microscopy was done at X40 magnification, and collagen volume fractions (CVF) were analyzed with ImageJ.

#### Immunohistochemical (IHC) staining

CD86 (Abcam; ab53004; 1:1000) and CD206 (Abcam; ab8918; 1:1000) IHC staining were done as previously described (17), but with few optimizations. Briefly, frozen sections were used; hence, the antigen retrieval step in the described experiment was skipped, and myocardial sections were fixed with 4% formaldehyde for 15 min prior to staining. Infiltrated CD86+ and CD206+ macrophages were observed at X40 magnification and quantified with ImageJ.

#### Oil Red O (ORO) staining

To investigate the metabolic state of the hearts, lipid depositions in myocardia were assessed by performing ORO staining described by the manufacturer (Solarbio; G1261). Lipid depositions were observed at X40 magnification and quantified with ImageJ.

#### Periodic Acid Schiff (PAS) staining

To examine the hearts’ metabolic state, glycogen and other polysaccharide contents of the myocardia were assessed by performing PAS staining described by the manufacturer (Solarbio; G1281). PAS-positive areas were observed at X40 magnification and quantified with ImageJ.

### Western blot

Hearts were washed with cold PBS, homogenized, and cocktails of RIPA buffer, protease, and phosphatase inhibitor (ratio 100:1:1) were added to extract proteins. Protein sample concentrations were normalized, electrophoresed on 10-12% gels, and transferred onto 0.45 μm PVDF membrane (Millipore Immobilon^®^-P; IPVH08100). Membranes were blocked with 2% BSA in TBST, and proteins of interest were blotted with the following antibodies: anti-HIF-1α (Proteintech; 20960-1-AP; 1:1000), anti-HIF-2α (Abcam; ab199; 1:1000), anti-Nrf2 (Proteintech; 16396-1-AP; 1:1000), anti-β_2_AR (Abcam; ab182136; 1:1000), anti-Scarb3 (Abcam; ab133625; 1:1000), anti-Slc2a1 (Abcam; ab115730; 1:1000), anti-GATA4 (Abcam; ab84593; 1:1000), GAPDH (Proteintech; 10494-1-AP; 1:1000) and HRP-conjugated Goat Anti-Rabbit IgG(H+L) (Proteintech; SA00001-2; 1:1000). Membranes were imaged using enhanced chemiluminescence (Tanon, China).

### Quantitative RT-PCR

mRNAs were isolated from myocardial macrophages with TRIzol™ Reagent (Invitrogen™; 15596026), cDNAs synthesized using a reverse transcription kit (FSQ107; Toyobo), and qPCR analysis was conducted by utilizing SYBR Green Master Mix (Q111-02; Vazyme) according to manufacturer instructions. The assessed macrophage metabolic genes (Gcdh, Adcd1, Acaa2, Decr1, Hsd17b4, Hadha, Cpt2, Etfb, Echdc2, Scarb3, mTOR, Slc2a1, Hk2, Ldha, Aldoc, Fbp1, Pgm2, Gpi1, Pgk1, and Pfkfb3) and their respective primer sequences are tabulated here (**Supplementary Table 1**). GAPDH was utilized as the housekeeping gene, and mRNAs fold changes were computed by the 2^−ΔΔCt^ method.

### Cardiopulmonary exercise test in humans

Before and after the application of OP intervention (6 cycles of 5 min occlusion with 130% of systolic pressure and reperfusion at 0 mmHg – alternating upper limb daily for 6 consecutive days), all human subjects were assessed by cardiopulmonary exercise testing (CPET) (Vyaire Medical; Vyntus^®^ CPX) on a bicycle ergometer (Stex Fitness; S25U) using the ramp 10 Watts protocol (10 W increment in workload per 1 min). Data analysis included the following physiological indexes; heart rate (HR), systolic (Psys) and diastolic (Pdia) pressures, expiratory reserve, inhalation and exhalation vital capacities, minute ventilation (V’E), carbon dioxide output (V’CO_2_), oxygen uptake (V’O_2_), oxygen pulse (V’O_2_/HR), metabolic equivalent of task (MET) and respiratory exchange ratio (RER). Additionally, oxygen saturation (SPO_2_) was measured with an ear sensor probe (Integrated Nonin™).

### Statistics

All results in this study are presented as mean ± standard error of the mean. Statistical analyses were done using GraphPad Prism (Software version 8.0.2). Unpaired t-test was used for comparing two groups, one-way ANOVA was used for comparing three or more groups, and two-way ANOVA was used for grouped data statistical analyses. P-values less than 0.05 were deemed statistically significant.

## Result

### OP preserves cardiac electric activity during hypobaric hypoxia

**Figure 1:**
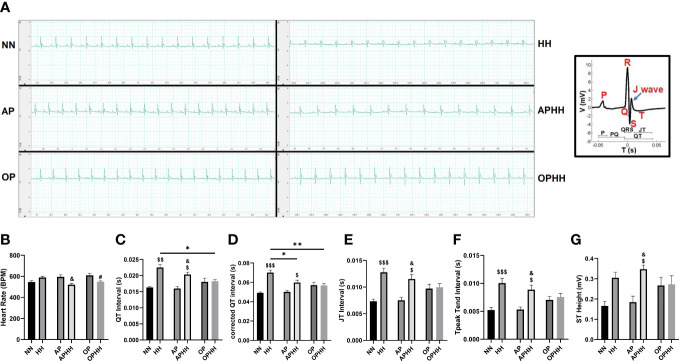
OP preserves cardiac electric activity during hypobaric hypoxia. **(A)** Representative electrocardiography (EKG) of Normobaric Normoxia (NN), Hypobaric Hypoxia (HH), Altitude Preconditioned (AP), Altitude Preconditioned before HH exposure (APHH), Occlusion Preconditioned (OP) and Occlusion Preconditioned before HH exposure (OPHH) mice. P wave: atrial depolarization; Q wave: Interventricular septum depolarization; R wave: Ventricular depolarization; S wave: Purkinje fibres depolarization; J wave: Early ventricular repolarization; T wave: End of ventricular repolarization. **(B-G)** Graphical presentation of EKG parameters including; Heart Rate (HR), QT Interval, corrected QT Interval (QTc), JT Interval, Tpeak Tend Interval, and ST Height. (n= 5-9 mice per experimental group). ^$^p<0.05, ^$$^p<0.01, ^$$$^p<0.001 HH vs NN; ^&^p<0.05 APHH vs AP; ^#^p<0.05 OPHH vs OP; *p<0.05, **p<0.01. Data are expressed as mean ± SEM. Data were analyzed using one-way ANOVA, followed by Tukey’s *post hoc* analysis.

Electrocardiograms (EKG) of mice post-HH exposure showed overt distortions in their cardiac electric activity (cEA) in comparison with the normobaric normoxia (NN) mice (control group). A detailed look at the EKG parameters revealed HH mice had modest increments in their heart rates (HRs) but with significant prolongations of QT, QTc, JT, and Tpeak-Tend intervals, and ST height, P duration, and R and T amplitudes (**Figures 1A–G**; **Supplementary Table 2**). Taken together, the aforementioned EKG alterations indicate that HH mice had severe arrhythmias resulting from chronic exposure to hypoxia. To determine the efficacy of OP’s impact on OPHH mice, we employed AP and APHH mice groups for comparison. The EKG data indicated that, similar to AP, OP prevents disruption of cEA with initial slight elevations of HRs and its normalization when exposed to HH. However, we uncovered that OP preserved cEA better than AP because unlike in OPHH mice – QT, QTc, JT, and Tpeak-Tend intervals, and ST height remained significantly increased in APHH compared to NN mice.

### OP mitigates myocardial hypertrophy, injury, and fibrosis during hypobaric hypoxia

**Figure 2:**
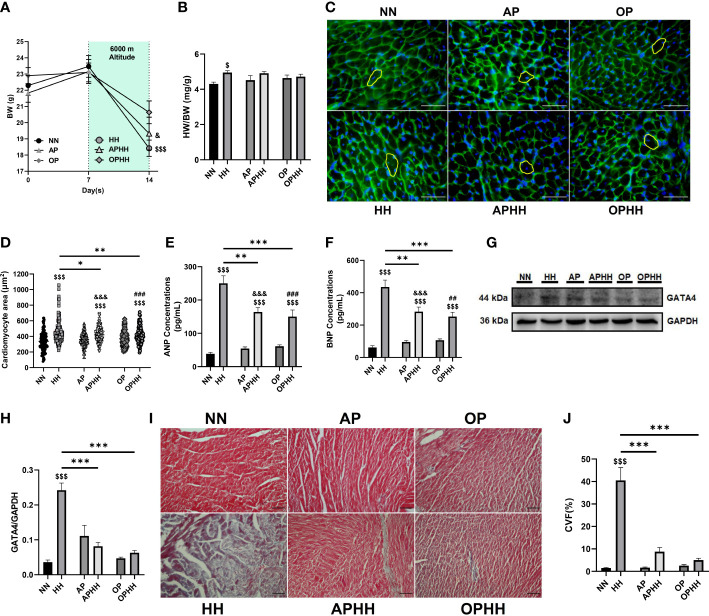
OP mitigates myocardial hypertrophy, injury, and fibrosis during hypobaric hypoxia. **(A)** Graphical representation of Body Weight (BW) trends of 14 days period by Normobaric Normoxia (NN), Hypobaric Hypoxia (HH), Altitude Preconditioned (AP), Altitude Preconditioned before HH exposure (APHH), Occlusion Preconditioned (OP) and Occlusion Preconditioned before HH exposure (OPHH) mice (n= 7-15 mice per experimental group). **(B)** Graphical presentation of Heart Weight (HW)/BW ratio (n= 5-10 mice per experimental group). **(C-H)** Indexes for cardiac hypertrophy assessment, including; Representative Wheat germ agglutinin (WGA) staining and Graphical presentation of Cardiomyocyte surface area (n=8-12 cells per section per 4-6 heart per group), Atrial natriuretic peptide (ANP) and Brain natriuretic peptide (BNP) concentrations (n=6-8 hearts per group), Representative Immunoblotting of GATA4 and its Graph plot (n= 3 hearts per group). ELISA were performed in triplicates. Immunoblots were performed in triplicates, and each blot band in the representative blot is an independent biological sample. **(I, J)** Representative Masson’s trichome staining and Graphical presentation of collagen volume fraction (CVF) showing the extent of fibrosis among experimental groups (n= 3-6 sections per 4,5 hearts per group). ^$^p<0.05, ^$$$^p<0.001 HH vs NN; ^&^p<0.05, ^&&&^p<0.001 APHH vs AP; ^##^p<0.01, ^###^p<0.001 OPHH vs OP; ^*^p<0.05, ^**^p<0.01, ^***^p<0.001. Data are expressed as mean ± SEM. Data were analyzed using one-way anova, followed by Tukey’s *post hoc* analysis.

Mice exposed to HH without any preconditioning exhibited significant body weight (BW) loss and increased heart weight/body weight ratio (**Figures 2A, B**), depicting cardiac hypertrophy. Next, cardiomyocyte surface area, atrial natriuretic peptide (ANP), brain natriuretic peptide (BNP), and GATA4 expressions were ascertained to validate the incidence of cardiac hypertrophy (**Figures 2C–H**). These indexes and biomarkers were substantially increased in HH mice. Also, the extent of ANP, BNP, and GATA4 upregulation in HH heart revealed the incidence of cardiac injury. Compared with HH and APHH mice, OPHH mice showed the least weight loss and moderate increases in the prior mentioned cardiac hypertrophy and cardiac injury indexes. In addition, utilizing trichrome staining, we observed massive fibrosis in HH hearts (**Figures 2I, J**). Meanwhile, like APHH, OPHH mice had only modest collagen depositions with no significant differences in comparison to the NN mice. These findings indicated that employing the OP intervention before exposure to chronic HH confers cardioprotection by mitigating excessive myocyte hypertrophy, injury, and myocardial fibrosis.

### OP induces adaptive immunomodulation and metabolic homeostasis during hypobaric hypoxia

**Figure 3:**
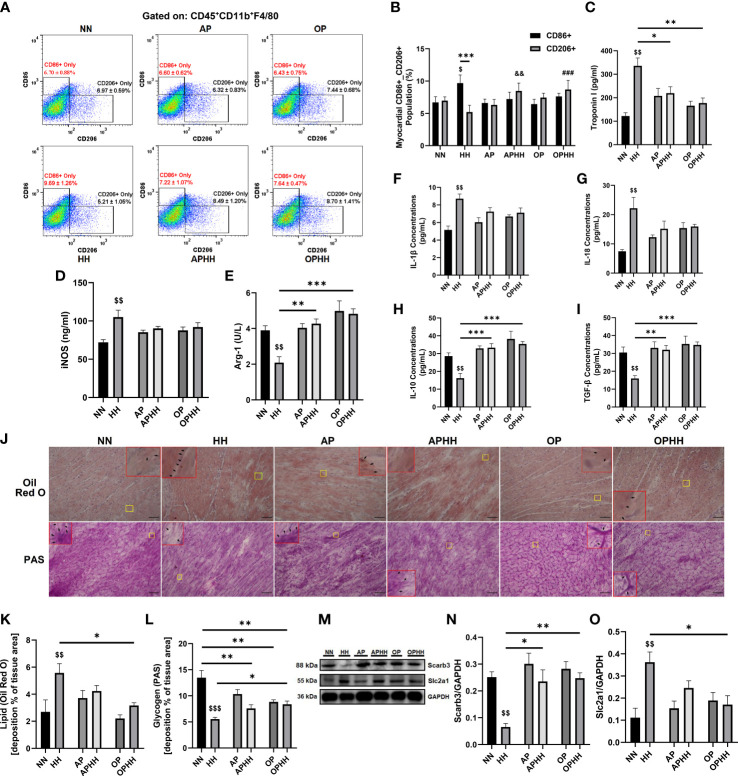
OP induces adaptive immunomodulation and metabolic homeostasis during hypobaric hypoxia. **(A, B)** Representative flow cytometry of myocardial macrophages gated on CD45^+^CD11b^+^F4/80 and Graphical plots of CD86^+^ and CD206^+^. Normobaric Normoxia (NN), Hypobaric Hypoxia (HH), Altitude Preconditioned (AP), Altitude Preconditioned before HH exposure (APHH), Occlusion Preconditioned (OP), and Occlusion Preconditioned before HH exposure (OPHH) mice hearts (n=4 hearts per group). ^$^p<0.05 vs NN_CD86+_; ^##^p<0.01, ^###^p<0.001 vs HH_CD206+_ **(C)** Graphical presentation of sera cardiac troponin I (cTnI) concentrations. **(D, E)** Inflammatory mediators; Inducible nitric oxide synthase (iNOS) and Arginase-1 (Arg-1) concentrations assessed by ELISA using myocardia lysates. **(F-I)** Inflammatory cytokines; Interleukin (IL)-1β, IL-18, IL-10, and transforming growth factor (TGF)-β concentrations assessed by ELISA using myocardia lysates. All ELISA were performed in triplicates (n= 5-8 mice per group). **(J-L)** Representative Oil Red O (ORO) and Periodic Acid Schiff (PAS) staining of myocardial sections and their respective graphical presentations showing lipid and glycogen depositions percentages (n=4-6 sections per 4-6 mice per group). Yellow outlined boxes are original myocardial portions and red outline boxes are their zoomed-in (5x) inserts to show positive stained area (indicated with black arrows). **(M-O)** Representative Immunoblotting of Scarb3 and Slc2a1 and their respective Graphical plots; each blot band in the representative blot is an independent biological sample (n= 3 hearts per group). ^$$^p<0.01, ^$$$^p<0.001 HH vs NN; ^&&^p<0.01 APHH vs AP; ^*^p<0.05, ^**^p<0.01, ^***^p<0.001. Data are expressed as mean ± SEM. Data were analyzed using one-way ANOVA, followed by Tukey’s *post hoc* analysis.

To understand how severe hypoxia affects immunoregulation in the myocardia, we investigated the phenotype of macrophages infiltrating the heart after chronic exposure to HH. We observed a substantial influx of CD86+ (proinflammatory) macrophages into the myocardial of HH mice, while the CD206+ (anti-inflammatory) populations were repressed (**Figures 3A, B**; **Supplementary Figures 2A-C**). Contrarily, CD206+ macrophages outnumbered the CD86+ cells when AP and OP interventions were applied prior to HH exposure. Remarkably, the degree of CD86+ macrophage infiltrations across all the groups corresponded to sera levels of the damage-associated molecular pattern (DAMP) – cardiac troponin I (cTnI) (**Figure 3C**). Further investigations assessed the concentrations of inflammatory mediators and cytokines in the hearts. The proinflammatory response mediator – iNOS, was overtly upregulated in HH mice but modestly in AP and OP hearts and without any significant alterations in APHH and OPHH hearts; meanwhile, the contrast was observed for the anti-inflammatory mediator, arginase (Arg)-1 (**Figures 3D, E**). Similarly, we found that proinflammatory cytokines (IL-1β and IL-18) were significantly upregulated in HH but only moderately in APHH and OPHH mice hearts (**Figures 3F, G**). Additionally, the systemic inflammatory mark (CRP) was prominently upregulation in HH but not in APHH and OPHH mice (**Supplementary Figure 2D**). However, like Arg-1, the anti-inflammatory cytokines (IL-10 and TGF-β) secretions were repressed in HH but modestly increased in APHH and OPHH hearts (**Figures 3H, I**). These findings demonstrated that just like AP, the OP intervention circumvents HH-induced myocarditis by minimizing cardiomyocyte necrosis and DAMP secretions – ultimately preventing the induction of hyperactive proinflammatory responses.

Furthermore, besides cardiac fibroblast activation, hypoxia-induced glycolysis shift orchestrates immune cells reprogramming toward proinflammatory phenotypes (18–20). Hence, the metabolic states of the infiltrated CD45^+^F4/80^+^CD11b^+^ cells and the entire myocardial were assessed. We observed that lipid metabolism-related gene expressions (Gcdh, Adcd1, Acaa2, Decr1, Hsd17b4, Hadha, Cpt2, Etfb, Echdc2, and Scarb3) in the macrophages isolated from HH hearts were mostly downregulated. In contrast, glycolysis-related genes (Slc2a1, Hk2, Ldha, Aldoc, Fbp1, Pgm2, Gpi1, Pgk1, and Pfkfb3) and the cellular metabolic regulator – mechanistic target of rapamycin (mTOR) mRNA levels were upregulated. Intriguingly, we found only modest downregulation of the lipid metabolism-related genes and slight increases in mRNA levels of the glycolysis-related and mTOR genes in CD45^+^F4/80^+^CD11b^+^ cells obtained from APHH and OPHH hearts (**Supplementary Figure 2E**). Consistently, Oil red O and PAS staining showed HH mice myocardial had abundant lipid depositions compared to the NN mice, while glycogen and other polysaccharide contents were substantially depleted. Meanwhile, OPHH mice myocardial revealed a balance constitution of lipid metabolism and glycolysis substrates, as similarly observed in APHH hearts (**Figures 3J–L**). These phenomena were validated by immunoblotting myocardial protein lysate, which revealed that HH hearts had decreased expression of the fatty acid transporter (Scarb3). In contrast, the glucose transporter protein (Slc2a1) was increased – indicating a glycolysis shift in HH hearts but not entirely in APHH and OPHH hearts (**Figures 3M–O**). Overall, these findings indicate that the OP intervention exerts immunomodulation by adaptively regulating metabolic shifts to prevent glycolytic reprogramming of macrophages towards proinflammatory phenotypes during HH.

### OP induces adaptive modulation of oxidative stress responses

**Figure 4:**
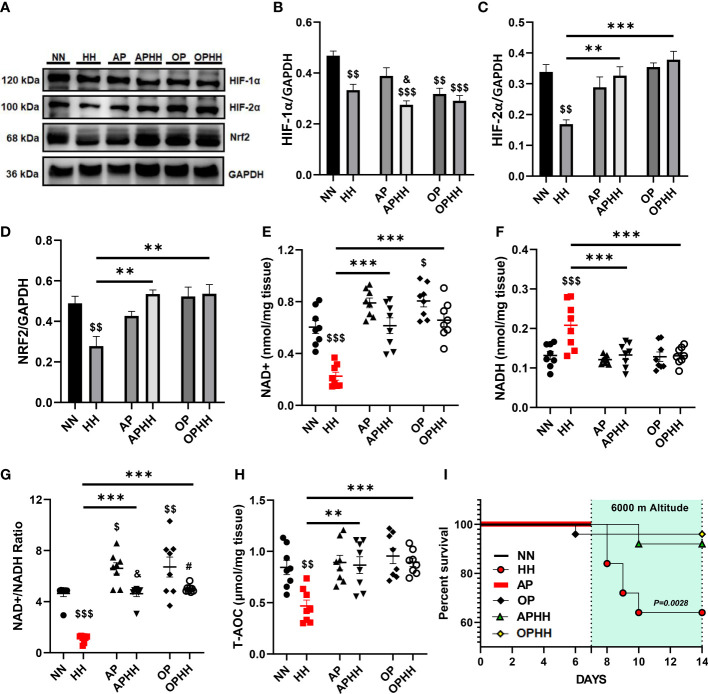
OP induces adaptive modulation of oxidative stress responses. **(A-D)** Representative Immunoblotting of Hypoxia-inducible factors (HIF)-1α, HIF-2α, and nuclear factor erythroid 2–related factor 2 (Nrf2), and their respective Graphical plots; each blot band in the representative blot is an independent biological sample (n= 4 hearts per group). Normobaric Normoxia (NN), Hypobaric Hypoxia (HH), Altitude Preconditioned (AP), Altitude Preconditioned before HH exposure (APHH), Occlusion Preconditioned (OP), and Occlusion Preconditioned before HH exposure (OPHH) mice hearts. **(E-H)** Antioxidants state indexes; Graphical plots of the concentrations of redox cofactors, Nicotinamide adenine dinucleotide (NAD)+hydrogen (NADH) and their ratio, as well as the Total antioxidant capacity (T-AOC) of the myocardia (n=8 hearts per group). **(I)** Graphical plot of survival data in Kaplan-Meier estimator (n=18 mice per group). ^$^p<0.05, ^$$^p<0.01, ^$$$^p<0.001 HH vs NN; ^&^p<0.05 APHH vs AP; ^#^p<0.05 OPHH vs OP; ^**^p<0.01, ^***^p<0.001. Data are expressed as mean ± SEM. Data were analyzed using one-way ANOVA, followed by Tukey’s *post hoc* analysis. Survival curves were analyzed with the Kaplan-Meier estimator.

Redox homeostasis (balance between ROS and antioxidants) signaling are major alterations occurring during chronic hypoxia (7). Hence, to elucidate the underlying mechanisms employed by the OP intervention during HH, we investigated its impact on oxidative stress regulators – hypoxia-inducible factors (HIF-1α and HIF-2α) and antioxidant response element-dependent genes regulator, nuclear factor erythroid 2–related factor 2 (Nrf2). Compared to the control group (NN), we observed significant decreases of HIF-1α expression in HH mice hearts and further sharp declines in the protein levels when AP or OP interventions were applied before HH exposure. Conversely, we observed that HIF-2α and Nrf2 expressions were reduced substantially in HH but modestly upregulated in APHH and OPHH mice hearts (**Figures 4A–D**). Nicotinamide adenine dinucleotide (NAD+) and NADH ratio are good predictors of the redox homeostasis state (21); as such, NAD+ and NADH contents in the heart were assessed. The outcomes demonstrated that AP and OP increased NAD+/NADH ratio modestly compared to NN. Secondly, HH hearts had a ~60% decrease in NAD+ and ~35% increase in NADH contents, thereby decreasing the NAD^+^/NADH ratio compared with NN, APHH, and OPHH hearts (**Figures 4E–G**). This indicated increased ROS with deficient antioxidant defenses in HH hearts but not in APHH and OPHH hearts which employed AP and OP interventions, respectively. The total antioxidant capacity assays (T-AOC) performed with myocardial lysates validated the prior statement. T-AOC of HH hearts reduced significantly but remained unaltered in APHH and OPHH, compared to NN hearts (**Figure 4H**). Thus, similar to AP, the OP intervention reinforces antioxidant responses to confer protection against hypoxia-induced oxidative stress damage – improving survival rates of OPHH compared to HH mice (**Figure 4I**).

### OPHH mice are resilient to HH-associated maladaptive behavioral outcomes

**Figure 5:**
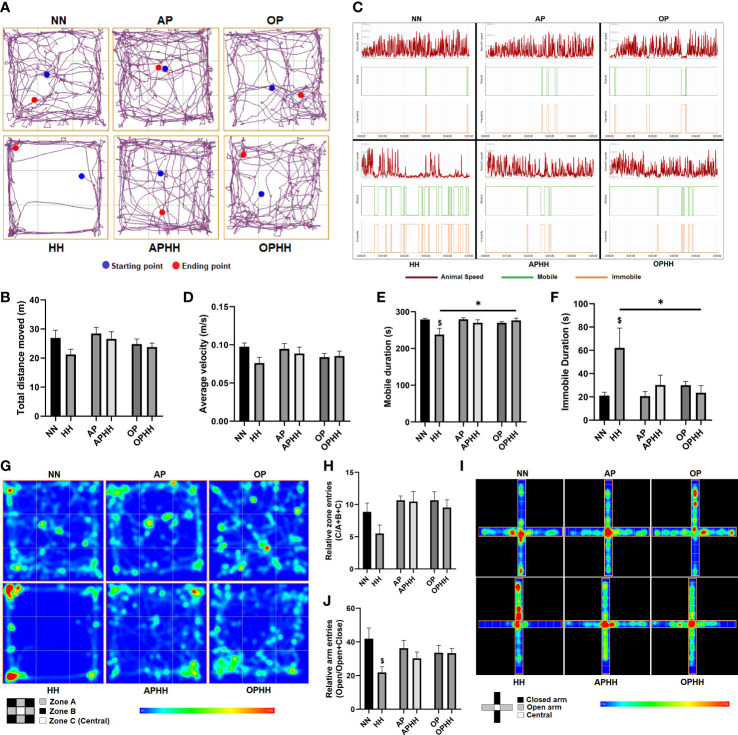
OPHH mice are resilient to HH-associated maladaptive behavioral outcomes. **(A-H)** Locomotive and exploratory behavioral assessment indexes from Open field test (OFT), including; Representative plots of Total distance moved and its graphical presentation, Representative plots of Average velocity, Mobile duration, and Immobile duration, and their graphical presentations and Representative heat-maps of Relative zone entries and its graphical presentation (n=6-10 mice per group). Normobaric Normoxia (NN), Hypobaric Hypoxia (HH), Altitude Preconditioned (AP), Altitude Preconditioned before HH exposure (APHH), Occlusion Preconditioned (OP), and Occlusion Preconditioned before HH exposure (OPHH) mice. **(I, J)** Anxiety-related and exploratory behavioral assessment indexes from Elevated plus maze (EPM), including; Representative heat-maps of Relative arm entries and its graphical presentation (n=6-10 mice per group). ^$^p<0.05 HH vs NN; *p<0.05. Data are expressed as mean ± SEM. Data were analyzed using one-way ANOVA, followed by Tukey’s *post hoc* analysis.

Since OP intervention prevented myocarditis and distortion of cEA in OPHH mice, we next tested whether it influenced behavioral outcomes. We observed that HH mice exhibited the most dullness among the experimental groups. Utilizing the open field test (OFT) to validate our observation, it was determined that the HH mice had the most decreases in locomotive function – with the least total distance moved, average velocity, and mobile duration compared to NN, APHH, and OPHH mice. Also, HH mice exhibited significant immobile duration in the OFT, but this was not the case for APHH and OPHH mice (**Figures 5A–F**). Thus, similar to AP in APHH, the OP intervention resulted in only modest reductions of locomotive functions in OPHH mice.

In addition, most HH mice were observed to restrict their movement to the peripheries (zone A) and corners (zone B) but avoided the central (zone C) of the OFT apparatus, thereby, had the least relative entries into zone C (**Figures 5G, H**) – a phenomenon shown to depict an increase in anxiety-related behaviors (22). Intriguingly, neither APHH nor OPHH mice exhibited the same exploratory behavior as HH mice, although they were all exposed to chronic hypoxia. The elevated plus maze (EPM) was used to confirm increased anxiety-related behaviors in HH mice. Most HH mice refrained from exploring the EPM apparatus’s open arms and mostly limited their movement to within the closed arm; hence, they had the least relative open arm entries (**Figures 5I, J**). Conversely, APHH and OPHH mice still demonstrated exploratory patterns in both open and closed arms similar to NN and the respective preconditioning groups. Taken together, the findings from OFT and EPM indicated that chronic hypoxia exposure increased susceptibility to developing anxiety-related behavior, as reported previously (22). Meanwhile, like AP to APHH, OP intervention makes OPHH mice resilient to hypoxia-induced negative behavioral outcomes.

### OP enhances respiratory and oxygen-carrying capacity in humans

**Figure 6:**
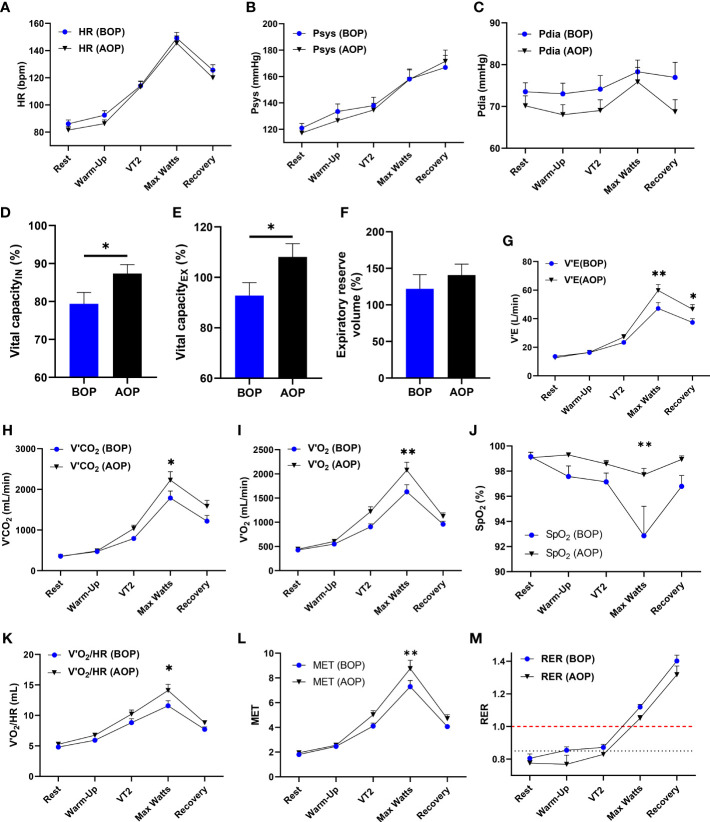
OP enhances respiratory and oxygen-carrying capacity in humans. **(A-M)** Cardiopulmonary Exercise Test (CPET) indexes for respiratory and oxygen carrying capacity in humans (n=14 human volunteers), Before occlusion preconditioning (BOP) and After occlusion preconditioning (AOP), including; Graphical plots of Heart Rates (HR), Systolic blood pressures (Psys), Diastolic blood pressures (Pdia), Vital capacity of inhalation (IN), Vital capacity of exhalation(EX), Expiratory reserve volume, Minute ventilation (V’E), Carbon dioxide output (V’CO_2_), Oxygen uptake (V’O_2_), Oxygen saturation (SpO_2_), Oxygen pulse (V’O_2_/HR), Metabolic equivalent of task (MET) and Respiratory exchange ratio (RER). Rest, Warm-up, Second ventilation threshold (VT2), Maximal workload (Max Watts) and Recovery are timepoints of interest during the CPET. *p<0.05, **p<0.01 AOP vs BOP. Data are expressed as mean ± SEM. Data were analyzed using an unpaired t-test for comparing two groups and two-way ANOVA for grouped analysis.

Still, in attempts to elucidate the underlying mechanism employed by the OP intervention, we assessed its impact on respiratory and oxygen-carrying capacity in humans. Cardiopulmonary exercise tests were performed on the healthy human subjects before (BOP) and after (AOP) the application of OP for 6 consecutive days. We found that HRs and diastolic and systolic blood pressures were slightly decreased in AOP compared to BOP (**Figures 6A–C**). Also, the inhalation and exhalation vital capacities were increased substantially while expiratory reserve volume elevated modestly in AOP compared to BOP (**Figures 6D–F**). The changes observed in the aforementioned indexes suggest that OP induces adaptive respiratory response and lowers the risk of pulmonary injuries and complications. Additionally, we found that after performing OP, minute ventilation (V’E), carbon dioxide output (V’CO_2_), oxygen uptake (V’O_2_), oxygen saturation (SpO_2_), oxygen pulse (V’O_2_/HR), and the metabolic equivalent of task (MET) were all significantly improved during the maximal workload (Max Watts) phase of exercising with the cycle ergometer (**Figures 6G–L**). Thus, OP enhanced the oxygen-carrying capacity and endurance in humans – an adaptation shown to mitigate the deleterious effects of severe hypoxia (23). Lastly, we observed that in AOP, the respiratory exchange ratio (RER) was lowered and took more time to reach ≥1.00 compared to BOP (**Figure 6M**). Consolidating our observation in the mice myocardia and macrophages, the lowered RER in AOP showed that OP induces mechanisms that mitigate the extent of metabolic shift to glycolysis.

### β_2_AR is implicated in OP-induced adaptive responses against hypobaric hypoxia

**Figure 7:**
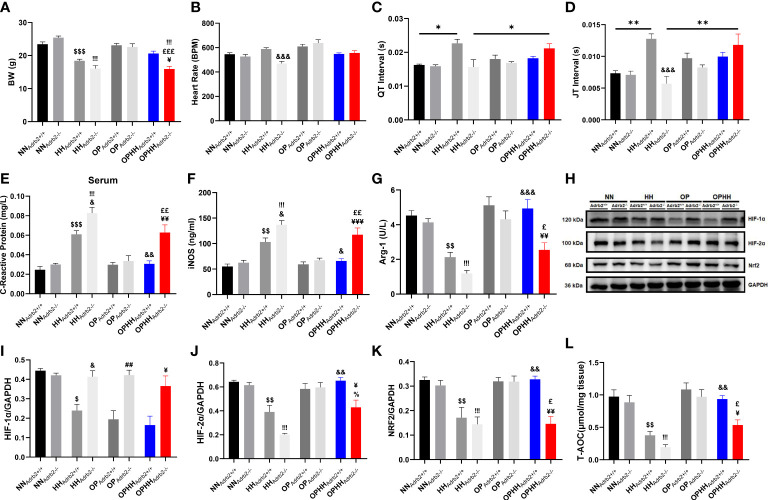
β_2_AR is implicated in OP-induced adaptive responses against hypobaric hypoxia **(A)** Graphical presentation of Body weight (BW) alteration trends among Wild type (Adrb2^+/+^) and β_2_AR knockout (Adrb2^-/-^) mice in experiment groups; Normobaric Normoxia (NN), Hypobaric Hypoxia (HH), Altitude Preconditioned (AP), Altitude Preconditioned before HH exposure (APHH), Occlusion Preconditioned (OP) and Occlusion Preconditioned before HH exposure (OPHH) (n=5-15 mice per group). **(B-D)** Graphical presentation of electrocardiogram (EKG) indexes, including; Heart Rate, QT Interval, and JT interval (n=5-9 mice per group). **(E-G)** Inflammatory biomarker; C-reactive protein, Inducible nitric oxide synthase (iNOS), and Arginase-1 (Arg-1) concentrations assessed by ELISA. Assays were performed in triplicates (n=4 mice per group). **(H-K)** Representative Immunoblotting of Hypoxia-inducible factors (HIF)-1α, HIF-2α, and nuclear factor erythroid 2–related factor 2 (Nrf2), and their respective Graphical plots; each blot band in the representative blot is an independent biological sample (n= 3 hearts per group). **(L)** Graphical plots of the concentrations of Total antioxidant capacity (T-AOC) of myocardia (n=4 hearts per group).^$^p<0.05, ^$$^p<0.01, ^$$$^p<0.001 vs NN_Adrb2+/+_; ^&^p<0.05, ^&&^p<0.01, ^&&&^p<0.001 vs HH_Adrb2+/+_; ^##^p<0.01 vs OP_Adrb2+/+_; ^¥^p<0.05, ^¥¥^p<0.01, ^¥¥¥^p<0.001 vs OPHH_Adrb2+/+_;^!!!^p<0.001 vs NN_Adrb2-/-_; ^%^p<0.001 vs HH_Adrb2-/-_; ^£^p<0.05, ^££^p<0.01, ^£££^p<0.001 vs OP_Adrb2-/-_. Data are expressed as mean ± SEM. Data were analyzed using two-way ANOVA.

The pleiotropic nature of β_2_AR makes it an essential mediator for most adaptive responses to stressful conditions in the heart and immunoregulation (14). Concordant with previous reports, we found β_2_AR overexpressed in HH compared to NN (24). Meanwhile, AP, APHH, OP, and OPHH hearts had modest upregulation of β_2_AR (**Supplementary Figure 3A, B**). By utilizing β_2_AR knockout (Adrb2^-/-^) mice, we explored the β_2_AR’s involvement in HH-induced myocarditis and arrhythmias and OP-induced cardioprotective against HH. Consistently, the OP intervention prevented significant body weight loss due to hypoxia in the OPHH_Adrb2+/+_ mice; however, the contrast was observed in the OPHH_Adrb2-/-_ mice (**Figure 7A**). While the HRs remained similar in OPHH_Adrb2+/+_ and OPHH_Adrb2-/-_, the EKG revealed arrhythmias in the latter (**Figure 7B**; **Supplementary Figure 3C**). We found that despite the occurrence of other forms of arrhythmias, the deletion of β_2_AR (in HH_Adrb2-/-_) circumvented the long QT, QTc, JT, and Tpeak-Tend intervals observed in HH_Adrb2+/+_. Intriguingly, this phenomenon was reverted when OP was applied to Adrb2-/- mice prior to HH exposure. The arrhythmias worsened in OPHH_Adrb2-/-_ as QT, QTc, JT, and Tpeak-Tend intervals prolongations and distortion of other EKG indexes were aggravated, compared to OPHH_Adrb2+/+_ (**Figures 7C, D**; **Supplementary Figure 3D, E** and **Supplementary Table 3**). These observed outcomes show that β_2_AR is involved in the OP-induced signaling cascades to preserve cEA during hypoxia.

Also, checking inflammatory markers, we determined the role of β_2_AR in OP-induced immunomodulation. At the baseline, proinflammatory responses (CRP and iNOS) were found further aggravated in HH_Adrb2-/-_ than in HH_Adrb2+/+_ mice. While the OP intervention significantly mitigated CRP and iNOS upregulations in OPHH_Adrb2+/+_, this phenomenon was abolished by β_2_AR obliteration in OPHH_Adrb2-/-_ mice (**Figures 7E, F**). Additionally, we found Arg-1 expression sustained in OPHH_Adrb2+/+_ but downregulated in the OPHH_Adrb2-/-_ (**Figure 7G**). Overall, the loss of adaptive immunoregulation in OPHH_Adrb2-/-_ compared to OPHH_Adrb2+/+_ mice suggests that β_2_AR-mediated signaling cascades are implicated in OP-induced immunomodulatory mechanisms.

Next, as oxidative stress-responsive genes were adaptively modulated in the wild-type OPHH mice heart, we examined whether β_2_AR participated in HIF-1α, HIF-2α, and Nrf2 expressions regulation. We observed that, unlike HIF-2α and Nrf2, HIF-1α was stabilized in HH_Adrb2-/-_ and also refractory to OP in Adrb2-/-, but not in the Adrb2+/+ hearts – suggesting that β_2_AR mediates the destabilization of HIF-1α (**Figures 7H–K**). Conversely, HIF-2α and Nrf2 expressions were sustained in OPHH_Adrb2+/+_ but not in OPHH_Adrb2-/-_ hearts, indicating that β_2_AR scaffolded the stabilization of these proteins. Further, we investigated the impact of these oxidative stress-responsive proteins alterations on the antioxidant capacity and found that obliteration of β_2_AR (in OPHH_Adrb2-/-_) weakened the cardioprotective antioxidant defense mechanisms induced by OP against hypobaric hypoxia (**Figure 7L**). Thus, these findings taken together shows that β_2_AR participates in multiple adaptive responses induced by OP to circumvent the adverse outcomes of chronic HH exposure.

## Discussions

The fear of experiencing altitude sickness deters civilian workers, hikers, tourists, and even defense personnel from going to places 2500 m above sea level for occupational or leisure purposes. Also, the possibility of HH at such altitudes to induce or aggravate myocarditis, arrhythmia, and ultimately heart failure due to adverse cardiac remodeling has made clinicians to advised those prone to cardiovascular complications and physiologically unprepared individuals against visiting high altitudes (25–27). Studies over the years have made attempts at elucidating the underlying pathomechanisms of altitude sickness and have generally demonstrated that the clinical manifestations observed are due to HH-induced maladaptive oxidative stress responses (imbalance between ROS and antioxidants), metabolic dysregulation and dampened anti-inflammatory defenses (7, 28, 29). Currently, AP and salidroside are the primary preventive therapeutic interventions being employed to circumvent or mitigate the adverse effects of HH exposure. Even so, inaccessibility to hypoxic chambers and unavailability of salidroside due to geographical limitations are the respective drawbacks of these interventions. Our study aimed to explore OP – which is potent in preventing hypoxia-induced damages (11–13), as an alternative therapeutic intervention for HH-induced myocarditis and arrhythmias. To ascertain the efficacy of OP intervention (in OPHH), its impacts on EKG, cardiac architecture, immunomodulation, oxidative stress regulation, and behavior outcomes were compared with APHH.

Concordant with previous studies (30, 31), we found that HH-induced tachycardia and prolongations of QT, QTc, JT, and Tpeak-Tend intervals, and ST height, P duration, and R and T amplitudes. Reportedly, these observations are because at HH; there is an increase in sympathetic activity, which triggers prolongation of repolarization, resulting in arrhythmia, heart failure, and sudden death (30). However, AP has been demonstrated to prevent significant disruption of cEA (32); consistently, our findings showed similar outcomes. Intriguingly, we observed that OPHH mice had fewer alterations in the EKG indexes than APHH mice compared to the NN mice. This led to the conclusion that OP preserved cEA modestly better than AP during HH exposure.

Furthermore, unlike in HH mice, we observed that BW losses were only modest, and the extent of cardiomyocyte hypertrophies, injury, and fibrosis were mitigated in APHH and OPHH hearts. Lippl et al. and others have similarly shown that at high altitudes, there is a loss of appetite hence the excessive BW (33, 34). Also, to compensate for oxygen demand, hypertrophy cascades are induced, resulting in excessive enlargement of cardiomyocytes and their apoptosis/necrosis, which in turn drives proinflammatory and fibrotic responses (33, 35). Interestingly, both AP and OP have been shown to lessen adverse cardiac remodeling during hypoxic or ischemic events, just as we observed – and it has also been suggested that both interventions might have similar underlying mechanisms (32, 36). While OP’s cardiac cardioprotection has been demonstrated mainly against ischemia/reperfusion injury, we show here for the first time that OP intervention is potent against HH-induced myocardial hypertrophy, injury, and fibrosis.

Myocarditis scaffolded by unresolved proinflammatory responses drives the maladaptive remodeling of the heart in HH (5); hence we investigated OP’s effect on immunomodulation. Typically observed at injured tissues or inflamed sites (37), we found massive infiltrations of proinflammatory (CD86+) macrophages in HH myocardia, whiles the reparative (CD206+) macrophage populations were significantly less. Also, inflammatory cytokines concentrations were altered in a similar fashion in the HH myocardia. In contrast, employing the OP intervention facilitated anti-inflammatory defenses while minimizing the proinflammatory responses in OPHH, as AP did in APHH. While we demonstrated OP’s immunoregulation in OPHH; Gorjipour et al.’s earlier works had shown similar observations where the OP intervention enhanced the elevation of IL-10 while downregulating the circulation IL-8 to confer cardioprotection in coronary artery bypass graft surgery (38). The metabolic state of inflammatory cells crucially influences their immune responses and functions (39); as such, we sought to ascertain the metabolic state of infiltrated macrophages and the entire myocardia in further investigations. Interestingly, we found that glycolysis-related genes had increased ~3 folds while that lipid metabolism-related genes were downregulated in macrophages isolated from HH hearts. These findings are consistent with metabolic shifts, which facilitate biased reprogramming of macrophages toward proinflammatory phenotypes (19, 39). Similarly, we observed that the entire HH myocardia had increased lipid deposition while glycogen and other polysaccharide contents were substantially depleted – all of which are consistent with glycolysis shift (40). Contrarily, OP intervention modulated metabolic homeostasis by preventing complete glycolysis shift (41), thereby impeding the reprogramming of macrophages towards proinflammatory phenotypes in OPHH, as similarly done by AP in APHH.

Also, disruption in redox homeostasis is a cofactor in HH-induced cardiac dysfunction (7), and our findings consolidated this fact, as HIF-1α, HIF-2α and Nrf2 expression were declining in HH mice hearts. Even so, it was observed that by employing OP intervention, HIF-2α and Nrf2 but not HIF-1α expressions were rescued and sustained in OPHH mice hearts. Similar outcomes were found in APHH mice hearts. These findings indicated that, like AP, OP stimulates adaptive oxidative stress regulation by reinforcing antioxidant responses. Consistently, OP has been shown to improve antioxidant defenses by enhancing NAD+ levels, which directly promotes Nrf2 antioxidant activities (42, 43). Next, we investigated OP’s impact on the NAD+/NADH ratio. We found that the OP intervention had increased the NAD+/NADH ratio and prevented significant decreases in OPHH hearts, which contributed to sustaining their T-AOC like in APHH hearts, while we observed declines in HH hearts. In line with our finding, Morris-Blanco et al. previously demonstrated that OP did increase NAD+/NADH ratio *via* protein kinase C epsilon (PKCε) in neuronal-glial (42), hence it is suggestive that similar mechanisms might be involved here. Overall, compared to HH, survival rates improved in OPHH and APHH.

Cardiovascular events promote anxiety-related behavior and vice versa; as such, it has become imperative to assess the behavioral outcomes of interventions targeted at improving cardiac health (44, 45). Typically, HH has been shown decrease locomotive functions while increasing the levels of anxiety and depression in humans (1). These were confirmed in the HH mice model as they had decreased total distance moved, average velocity, and mobile duration and mostly refrained from the central zone and open arms in the OFT and EPM apparatuses, respectively, during their locomotive and exploratory activities. Contrarily, like APHH mice, OPHH mice combed throughout zones and arms of the OFT and EPM apparatuses – indicating that similar to AP (22), the OP intervention makes mice resilient to HH-induced anxiety-related behavior and decreased locomotive functions.

To have prevented HH-induced cardiac remodeling, it was hypothesized that the OP intervention must have induced mechanisms to facilitate adequate tissue oxygenation. Surprisingly and in validating our hypothesis, CPET parameters in AOP showed enhanced respiratory and oxygen-carrying capacity and endurance in humans as V’E, V’CO_2_, V’O_2_, SpO_2_, V’O_2_/HR, and MET were all substantially improved at Max Watts (Maximal workload). Concordantly, it has been shown that OP mitigates declines in regional oxygenation to confer cardioprotection when exposed to HH (46). Also, OP modestly delayed the time for RER=1 in AOP compared to BOP; hence, indicating that the intervention induces mechanisms that mitigate the extent of metabolic shift to glycolysis, just as shown earlier and reported by others (41).

Lastly, we had previously demonstrated the adaptive roles of β_2_AR in cardioprotection and immunoregulation during stressful conditions (14); hence we sought to investigate its implication in OP-induced cardioprotective against HH. We observed that HH mice characterized with arrhythmias had β_2_AR expressions drastically increased in their hearts. Consistent with our observation, Lang et al. demonstrated that the overexpression of β_2_AR significantly increases the predisposition to the occurrence of arrhythmias (47). Surprisingly, β_2_AR deletion prevented long QT, QTc, JT, and Tpeak-Tend intervals in HH_Adrb2-/-_, mimicking the effect of β-blockers in preventing long QT syndrome (48). Meanwhile, most of the adaptive responses we observed in OPHH_Adrb2+/+_ mice were abolished in OPHH_Adrb2-/-_ mice as their BW losses were substantial, arrhythmia worsened, proinflammatory responses heightened against anti-inflammatory responses, and antioxidant defenses declined significantly. Consistent with our observations, carvedilol (a β-blockers) was shown to have abolished the cardioprotection conferred by OP during cardiac surgery (49).

In conclusion, by preserving cEA, mitigating cardiac remodeling, facilitating adaptive immunomodulation and oxidative stress responses, sustaining homeostasis in metabolic shifts, causing resilient against anxiety-related behaviors, and enhancing respiratory and oxygen-carrying capacity, OP demonstrates as a potent alternative therapeutic intervention for preventing HH-induced adverse effects on cardiac and overall health. Even so, OP is not recommended as an intervention for individuals on β-blocker medications prior to their visit to high altitudes/HH environments, as these medications blunt the cardioprotection conferred by the intervention and conversing aggravates myocarditis and arrhythmias. Lastly, immunoregulatory and metabolism homeostasis induced by OP is suggestive of its potential to ameliorate the progression of other inflammatory, metabolic and oxidative stress-related diseases.

## Data availability statement

The original contributions presented in the study are included in the article/supplementary material. Further inquiries can be directed to the corresponding authors.

## Ethics statement

The studies involving human participants were reviewed and approved by Ethics Committee of the Xuzhou Medical University. The patients/participants provided their written informed consent to participate in this study. The animal study was reviewed and approved by Experimental Animal Centre of Xuzhou Medical University and the Animal Ethics Committee of the Xuzhou Medical University.

## Author contributions

GKA and HS conceived and designed the project. GA, RM, RR, AA, MN, and PW performed animal models. GA, RM, RR, AA, and MN performed most experiments and analyzed data. GA, RR, AA, MN, PW, SA and JA-A performed assays. Cardiopulmonary Exercise Test was facilitated by JX, and FW, GA, LF, and JX conducted examinations and analyses. GA, SA, and XL performed flow cytometry experiments and analysis. GA, YL, and RM conducted behavioral experiments and analyses. GA wrote the manuscript with input from all authors and was approved by JX and HS. All authors contributed to the article and approved the submitted version.
