# Supplementary material for: Occlusion preconditioned mice are resilient to hypobaric hypoxia-induced myocarditis and arrhythmias due to enhanced immunomodulation, metabolic homeostasis, and antioxidants defense
Source: Front Immunol. 2023 Feb 15;14:1124649. doi: 10.3389/fimmu.2023.1124649 (PMC9975755; doi:10.3389/fimmu.2023.1124649)
Supplement: Supplementary file 1 [file DataSheet_1.docx]

**SUPPLEMENTAL DATA**

**Fig. S1**

A


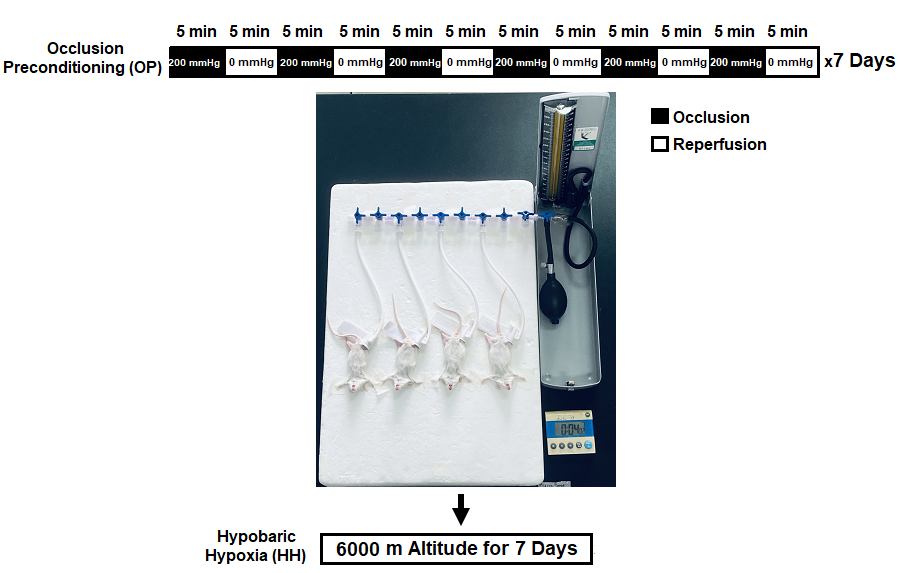


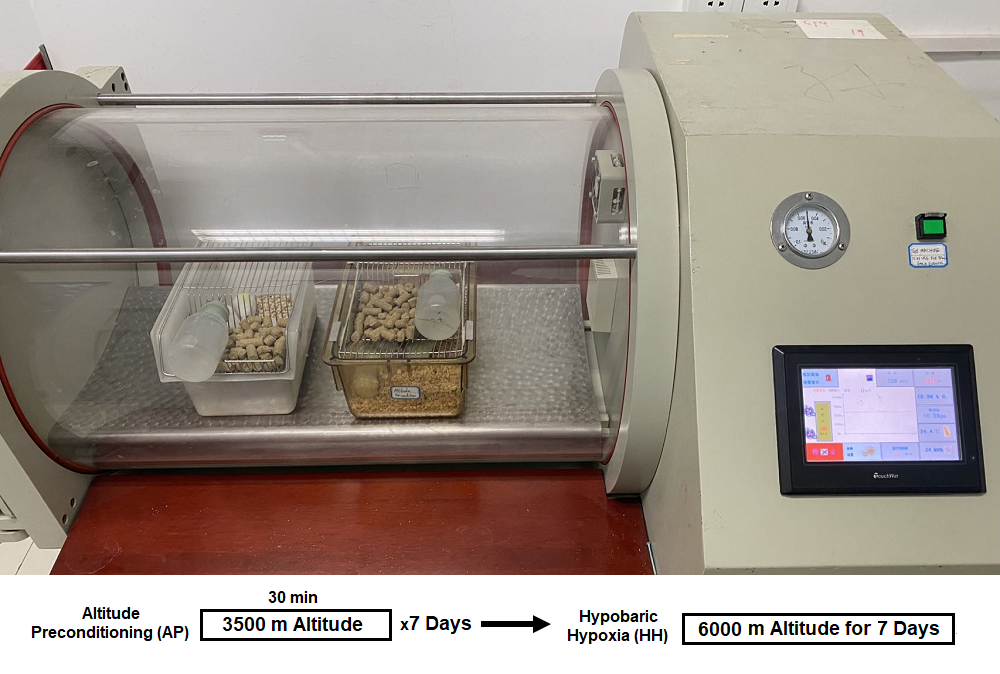


B

**Supplementary Figure 1: A)** Schematic and experimental set-up for Occlusion Preconditioning (OP) prior for exposure to Hypobaric Hypoxia (HH). **B)** Experimental set-up (Hypoxic Chamber) for Altitude Preconditioning (AP) or HH modelling.

| # | **Gene Name** | **Forward Sequence** | **Reverse Sequence** |
| --- | --- | --- | --- |
| 1 | Gcdh | GACAGTGGCTACAGGTCGATGA | GGCTCTGTAAGTCCAAAGCAGC |
| 2 | Abcd1 | TCCTGTCTGGAGGTGAGAAGCA | GCCTTCCACATCAATGCTCACG |
| 3 | Acaa2 | TCTGCTGGCAAAGTTCCACCTG | ACAGAGCCTGTTGAGGGTAAGG |
| 4 | Decr1 | CCAGTGTGTGATAGCCAGCAGA | TCAGGATCTCGAACATCACACCG |
| 5 | Hsd17b4 | TGGCTTTGCCATGAGAGCTGTG | CTGATTCCGCTTTCTGACGATGG |
| 6 | Hadha | GTTTGAGGACCTCGGTGTAAAGC | GAGAGCAGATGTGTTGCTGGCA |
| 7 | Cpt2 | GATGGCTGAGTGCTCCAAATACC | GCTGCCAGATACCGTAGAGCAA |
| 8 | Etfb | GGCTGAAAAGGAGAAAGTGGACC | GTCACCTGAGAGGCGAATGTAC |
| 9 | Echdc2 | GGCAAAGTTGCCATCGACAGAG | GTTTCTCCCTGAAGGCTGCCAT |
| 10 | Scarb3 (CD36) | GGACATTGAGATTCTTTTCCTCTG | GCAAAGGCATTGGCTGGAAGAAC |
| 11 | mTOR | AGAAGGGTCTCCAAGGACGACT | GCAGGACACAAAGGCAGCATTG |
| 12 | Slc2a1 | GCTTCTCCAACTGGACCTCAAAC | ACGAGGAGCACCGTGAAGATGA |
| 13 | Hk2 | CCCTGTGAAGATGTTGCCCACT | CCTTCGCTTGCCATTACGCACG |
| 14 | Ldha | ACGCAGACAAGGAGCAGTGGAA | ATGCTCTCAGCCAAGTCTGCCA |
| 15 | Aldoc | CACTCAATGCCTGGAGAGGACA | CGCCATCTCCACTGCCTTCATA |
| 16 | Fbp1 | TGCTGAAGTCGTCCTACGCTAC | TTCCGATGGACACAAGGCAGTC |
| 17 | Pgm2 | AGCCAATGACCCAGATGCTGAC | AGCCAATGACCCAGATGCTGAC |
| 18 | Gpi1 | CCATCAAGGTGGACGGCAAAGA | CCGTGATGGATTTGCCAGTGTAC |
| 19 | Pgk1 | GATGCTTTCCGAGCCTCACTGT | ACCAGCCTTCTGTGGCAGATTC |
| 20 | Pfkfb3 | TCATCGAGTCGGTCTGTGACGA | CATGGCTTCTGCTGAGTTGCAG |
| 21 | Gapdh | CATCACTGCCACCCAGAAGACTG | ATGCCAGTGAGCTTCCCGTTCAG |

**Table S1: qPCR Primer Pair**

**Table S2: Electrocardiography Data**

| **Parameters** | **NN** | **HH** | **AP** | **APHH** | **OP** | **OPHH** |
| --- | --- | --- | --- | --- | --- | --- |
| RR Interval (s) | 0.1100 ± 0.0055 | 0.1042 ± 0.0056 | 0.1015 ± 0.0083 | 0.1156 ± 0.0063 | 0.09896 ± 0.0093 | 0.1035 ± 0.0055 |
| PR Interval (s) | 0.03235 ± 0.00158 | 0.03252 ± 0.0043 | 0.03155 ± 0.00300 | 0.03338 ± 0.00200 | 0.03060 ± 0.00258 | 0.03315 ± 0.00243 |
| P Duration (s) | 0.01014 ± 0.00162 | 0.00643 ± 0.00054 | 0.00848 ± 0.00229 | 0.00762 ± 0.00155 | 0.00825 ± 0.00191 | 0.00791 ± 0.00182 |
| QRS Interval (s) | 0.00895 ± 0.00055 | 0.00796 ± 0.00115 | 0.00888 ± 0.00060 | 0.00879 ± 0.00067 | 0.00831 ± 0.00167 | 0.00830 ± 0.00075 |
| P Amplitude (mV) | 0.1519 ± 0.031 | 0.1654 ± 0.027 | 0.1529 ± 0.037 | 0.1466 ± 0.024 | 0.1124 ± 0.036 | 0.1560 ± 0.051 |
| Q Amplitude (mV) | 0.0350 ± 0.0248 | 0.0362 ± 0.0132 | 0.0325 ± 0.0198 | 0.0274 ± 0.0077 | 0.0270 ± 0.0211 | 0.0153 ± 0.0218 |
| R Amplitude (mV) | 1.270 ± 0.1441 | **0.6788 ± 0.1701** | 1.183 ± 0.3674 | 0.9925 ± 0.1621 | 1.441 ± 0.3633 | 1.428 ± 0.2570 |
| S Amplitude (mV) | -0.3356 ± 0.1445 | -0.2940 ± 0.2381 | -0.4198 ± 0.09968 | -0.3839 ± 0.1509 | -0.2846 ± 0.09965 | -0.5029 ± 0.3146 |
| T Amplitude (mV) | 0.3893 ± 0.09054 | 0.1322 ± 0.2318 | 0.4337 ± 0.07956 | 0.4925 ± 0.1062 | 0.5070 ± 0.1537 | 0.4770 ± 0.1411 |

Normobaric Normoxia (NN), Hypobaric Hypoxia (HH), Altitude Preconditioned (AP), Altitude Preconditioned before HH exposure (APHH), Occlusion Preconditioned (OP) and Occlusion Preconditioned before HH exposure (OPHH) mice. Data are expressed as mean ± standard deviation.

**BOLD** indicates significant (*p-*value<0.05) vs NN

**Fig. S2**

A


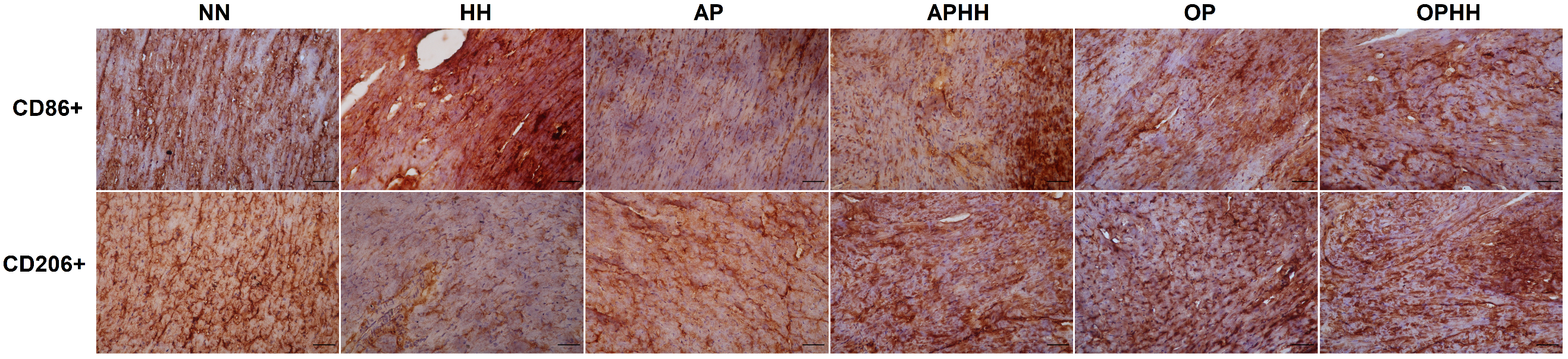

C

B

D

E

**Figure S2. OP induces adaptive immunomodulation and metabolic homeostasis during hypobaric hypoxia. A-C)** Representative Immunohistochemical (IHC) staining of CD86^+^ and CD206^+^ myocardial sections and their graphical plots depicting extent of infiltration (n=4-6 sections per 4-7 hearts). **D)**  Graphical presentation of sera levels of C-reactive protein concentrations assessed by ELISA in triplicates (n=6-8). **E)** Relative mRNA levels of metabolic responsive genes Gcdh, Adcd1, Acaa2, Decr1, Hsd17b4, Hadha, Cpt2, Etfb, Echdc2, Scarb3, Slc2a1, Hk2, Ldha, Aldoc, Fbp1, Pgm2, Gpi1, Pgk1, and Pfkfb3 (n=3). *p<0.05, **p<0.01, ***p<0.001 HH *vs* NN; $$$p<0.001 *vs* HH. Data are expressed as mean ± SEM. Data were analyzed using one-way ANOVA, followed by Tukey’s post hoc analysis

B

A

**Fig. S3**


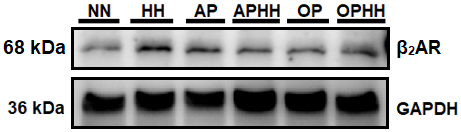


C


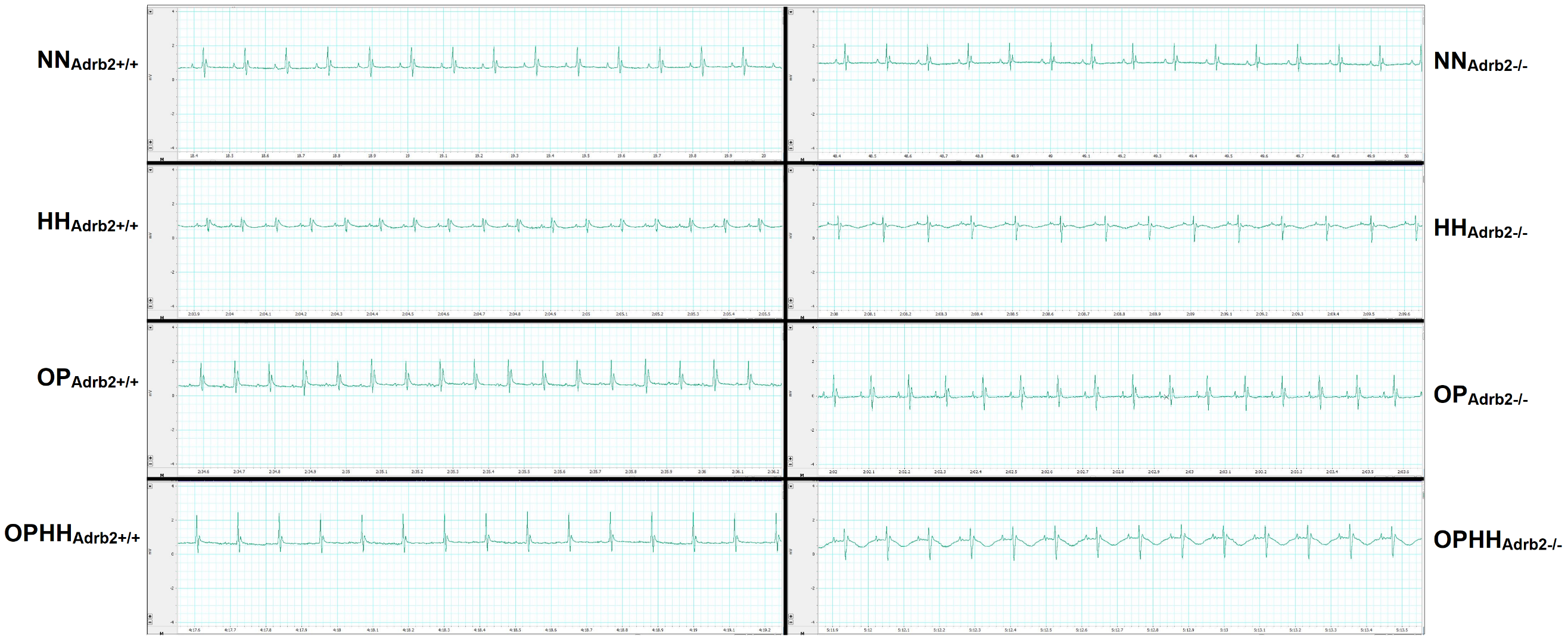


E

D

**Figure S3. β2AR is implicated in OP-induced adaptive responses against hypobaric hypoxia A,B)** Representative immunoblotting and graph plot of β_2_-Adrenergic Receptor (β_2_AR) expression. (n= 4 hearts per group). *p<0.05; $p<0.05, $$$p<0.001 HH vs NN. **C)** Representative electrocardiography (EKG) of β2AR knockout (Adrb2-/-) and Wild type (Adrb2+/+) mice in the following experimental groups; Normobaric Normoxia (NN), Hypobaric Hypoxia (HH), Altitude Preconditioned (AP), Altitude Preconditioned before HH exposure (APHH), Occlusion Preconditioned (OP) and Occlusion Preconditioned before HH exposure (OPHH). **D,E)** Graphical presentation of EKG parameters including; corrected QT Interval (QTc) and Tpeak Tend Interval and ST Height. (n= 5-9 mice per experimental group). &&&p<0.001 *vs* HH_Adrb2+/+_; **p<0.01. Data are expressed as mean ± SEM. Data were analyzed using two-way ANOVA.

**Table S3: Electrocardiography Data on β_2_AR knockout (Adrb2^-/-^) and Wild type (Adrb2^+/+^)**

| **Parameters** | **NN_Adrb2+/+_** | **NN_Adrb2-/-_** | **HH_Adrb2+/+_** | **HH_Adrb2-/-_** | **OP_Adrb2+/+_** | **OP_Adrb2-/-_** | **OPHH_Adrb2+/+_** | **OPHH_Adrb2-/-_** |
| --- | --- | --- | --- | --- | --- | --- | --- | --- |
| RR Interval (s) | 0.1099 ± 0.004720 | 0.1152 ± 0.007069 | 0.1020 ± 0.005299 | **0.1285 ± 0.01077** | 0.1015 ± 0.01063 | 0.09460 ± 0.008669 | 0.1033 ± 0.005752 | 0.1072 ± 0.006290 |
| PR Interval (s) | 0.03164 ± 0.002523 | 0.02715 ± 0.004108 | 0.03265 ± 0.005213 | 0.02859 ± 0.003660 | 0.03152 ± 0.003186 | 0.02814 ± 0.0008444 | 0.03455 ± 0.002583 | 0.03030 ± 0.001507 |
| P Duration (s) | 0.009234 ± 0.002569 | 0.006899 ± 0.001198 | 0.01118 ± 0.005840 | 0.008311 ± 0.000330 | 0.007618 ± 0.001672 | 0.008272 ± 0.000719 | 0.008122 ± 0.002169 | 0.007080 ± 0.001154 |
| QRS Interval (s) | 0.008993 ± 0.000462 | 0.008797 ± 0.0008478 | 0.008569 ± 0.001938 | 0.01007 ± 0.002597 | 0.008302 ± 0.002062 | 0.008635 ± 0.000409 | 0.008473 ± 0.000854 | 0.009377 ± 0.000914 |
| P Amplitude (mV) | 0.1868 ± 0.02776 | 0.1901 ± 0.03565 | 0.1592 ± 0.04371 | 0.1579 ± 0.02538 | 0.1221 ± 0.03865 | 0.2016 ± 0.06728 | 0.1509 ± 0.05128 | 0.2145 ± 0.05735 |
| Q Amplitude (mV) | 0.03496 ± 0.02491 | 0.01992 ± 0.01335 | 0.04761 ± 0.01678 | -0.04629 ± 0.1683 | 0.02116 ± 0.04160 | 0.03650 ± 0.007987 | 0.01885 ± 0.02278 | 0.04769 ± 0.01647 |
| R Amplitude (mV) | 1.252 ± 0.1240 | 0.9672 ± 0.1495 | **0.7572 ± 0.2006** | **0.3925 ± 0.1725** | 1.417 ± 0.4055 | 1.154 ± 0.1281 | 1.415 ± 0.2866 | 0.8909 ± 0.2342 |
| S Amplitude (mV) | -0.3129 ± 0.1195 | -0.4084 ± 0.1987 | -0.4129 ± 0.2021 | -0.6037 ± 0.3383 | -0.2547 ± 0.1076 | -0.7929 ± 0.3632 | -0.5649 ± 0.3106 | -0.8318 ± 0.3169 |
| T Amplitude (mV) | 0.3826 ± 0.07635 | 0.4395 ± 0.04527 | 0.1728 ± 0.3195 | 0.1180 ± 0.03005 | 0.4955 ± 0.1753 | 0.5915 ± 0.09246 | 0.4735 ± 0.1536 | 0.2578 ± 0.09651 |
| ST Height (mV) | 0.1662 ± 0.04986 | 0.1799 ± 0.05644 | 0.3048 ± 0.08590 | 0.03348 ± 0.04406 | 0.2669 ± 0.1202 | 0.2820 ± 0.08629 | 0.2728 ± 0.1030 | 0.2333 ± 0.08548 |

Normobaric Normoxia (NN), Hypobaric Hypoxia (HH), Altitude Preconditioned (AP), Altitude Preconditioned before HH exposure (APHH), Occlusion Preconditioned (OP) and Occlusion Preconditioned before HH exposure (OPHH) mice. Data are expressed as mean ± standard deviation.

**BOLD** indicates significant (*p-*value<0.05) vs NN_Adrb2+/_
